# How project-based learning improves EFL speaking in Chinese higher vocational colleges: a serial mediation study

**DOI:** 10.3389/fpsyg.2026.1927310

**Published:** 2026-10-20

**Authors:** Shuzhi Fan

**Affiliations:** School of General Education, City University of Zhengzhou, Zhengzhou, China

**Keywords:** autonomous motivation, collaborative engagement, English speaking competence, project-based learning, serial mediation

## Abstract

English-speaking competence remains a persistent challenge for Chinese EFL learners, and project-based learning has been advocated as a remedy, though existing evidence yields mixed effects and rarely specifies the psychological mechanisms or sub-skill profiles driving the variation. This explanatory sequential mixed-methods quasi-experimental study examined a 12-week project-based learning intervention with a 6-week delayed posttest in Chinese higher vocational college students (*N* = 179; PBL *n* = 93, CTL *n* = 86), testing autonomous motivation and collaborative engagement as serial mediators of English-speaking competence. Speaking was rated on a four-dimensional rubric covering fluency, accuracy, complexity, and pragmatic-interactional competence, with inter-rater intraclass coefficients of 0.76 to 0.87; serial mediation was estimated using PROCESS Model 6, and inductive thematic analysis of post-intervention interviews probed the operating mechanisms within project groups. Composite speaking showed a large condition advantage at the immediate posttest (*d* = 1.07), concentrated on fluency (*d* = 0.82) and pragmatic-interactional competence (*d* = 0.68), modest on complexity (*d* = 0.48), and not statistically detectable on accuracy. Retention 6 weeks later was differential, with motivation and engagement retaining 88 and 78% of their immediate gains while composite speaking competence retained 93%. The serial indirect path through engagement was significant whereas the motivation-only path was not, indicating that engagement carries the effect and motivation reaches speaking only through engagement. Qualitative themes located the active mechanism in group accountability while revealing compressing dynamics including free-riding, conversational dominance, and L1 slippage under time pressure. These findings imply that the active ingredient of project-based learning is the psychological condition it creates for learners rather than the task choreography, and that instructional design should center on supporting learners’ needs for autonomy, competence and relatedness, and specifically on the group accountability structures through which motivation becomes speaking.

## 1 Introduction

Speaking proficiency lags behind reading and writing abilities among Chinese college students, as the speaking ability has been identified by the learners themselves as the hardest skill to acquire (Huang et al., 2022). The skills of listening, reading and writing, which are often tested in exam situations, usually develop before speaking becomes possible in conversational settings. In other words, the problem does not lie in poor language proficiency but in psychological and interpersonal factors relating to its usage. A large number of students who score high in writing tests often have little involvement in oral communication due to nervousness and a lack of readiness to converse (Bai, 2023).

The project-based learning technique has become popular in the field of education because of its learner-focused and inquiry-oriented nature, which means that learners take part in real-life activities and use language meaningfully (Guo et al., 2020). The technique has proved to be more successful than the conventional technique, although the level of success depends on the subject, the period of activity, and the type of group (Zhang and Ma, 2023). Regarding the sphere of teaching foreign languages, project-based learning has a positive effect on the motivation and involvement of students in speaking classes, but there are some exceptions to particular issues (Zhang and Lin, 2025; Zhong et al., 2025). It is possible to increase motivation by using project-based methodologies, without substantially affecting achievement (Yang et al., 2025). This dissociation suggests that the effect of motivation appears before any change in performance takes place. The importance of the difference lies in the fact that methods of increasing motivation without improving performance require different types of assistance than other methods.

What remains unresolved in this line of research is not whether project-based learning affects oral performance but rather how this takes place. Though empirical research conducted in the language classroom suggests that such effects exist, few studies specify the psychological processes whereby classroom environments shape speech, nor does correlational design prove motivation as an intervening factor (Sánchez-García and Reyes-de-Cózar, 2025). Current studies consider oral language proficiency as one construct even though research findings have suggested that different dimensions of oral language proficiency develop at different rates (Wang et al., 2025). In addition, delayed assessment does not occur often, which makes it impossible to determine retention effect from novelty effect. Existing evidence on project-based speaking instruction is drawn almost exclusively from university or general college samples, whereas higher vocational college EFL learners—a large yet comparatively under-researched population in China—remain largely unexamined.

In the current research study, an integration of all these factors is attempted. Project-based learning is not considered to be a technique that needs validation but is regarded to be a psychological process that needs to be analyzed with respect to the mediation role of autonomy and collaboration in project-based learning in affecting learners’ speaking skills, the differences of such effects among various speaking skills, and how long these effects last after the completion of the process. With the relationship between the outcome and psychological aspects of motivation and cooperation being taken into consideration, the process of speaking is examined through certain skills, and the study extends far beyond the teaching phase. As a result, the research problem moves from the effectiveness of the approach to its mechanisms and influence on certain aspects of speaking and duration of that influence. The use of a quasi-experimental approach integrating elements of both quantitative and qualitative research enables estimation of such relationships and interpretation of the dynamics involved in collaboration for beneficiary determination.

## 2 Theoretical framework and hypotheses

### 2.1 Self-determination theory and the motivational pathway

The most plausible theory in support of how a project-based learning setting could affect how learners speak is Self-Determination Theory (SDT). As per the tenets of SDT, optimal motivation stems from the fulfillment of three psychological needs, namely autonomy, competence, and relatedness. The environment which satisfies these needs helps in a transition from external motivation to autonomy and self-motivation on the motivational continuum (Ryan and Deci, 2000). Meeting these needs increases autonomous motivation as well as the engagement that comes with it (Guay, 2022). A project-based learning classroom provides for all of the above mentioned needs: students gain autonomy through making decisions in relation to project development, competence through engaging in realistic activities, and relatedness through collaborating for a common outcome. Once all these needs are satisfied, the ensuing engagement and motivation bring about enhancement of speaking performance in English among Chinese university learners of English (Wang et al., 2024). It is not only the presence of projects but autonomous motivation which mediates the effect of intervention on speaking outcomes.

*H1*: Learners in the project-based condition make larger gains in autonomous motivation than learners taught through conventional instruction.

### 2.2 Collaborative learning and the engagement pathway

Another avenue is concerned with the behavior of learners in group settings. Collaborative learning assumes that the acquisition of knowledge is a constructive process whereby small group interactions act as the mode for the joint construction of meaning (Vygotsky and Cole, 1978). Working collaboratively on a joint task enables learners to make the behavioral and cognitive commitments involved in engagement, and group work in college English classes increases learner engagement because of the peer relations generated therein (Li, 2025). Motivation and cooperation depend on each other, and are not two separate concepts. Students with autonomous motivation cooperate more easily, take conversational risks, and persevere in their project in case of its stagnation. Thus, cooperation becomes possible because of motivation rather than a wholly separate route. Collaboration is not without its problems associated with individual differences. Group activities may result in unequal sharing, and such problems like free-riding and imbalance of participation in groups that are unbalanced negatively impact the possible advantages for students who need more practice with oral communication (Adesina et al., 2023). These problems are inherent in the process itself rather than just random disturbances, and form the basis of the qualitative part of the research.

*H2*: Learners in the project-based condition report higher collaborative engagement than their counterparts in conventional classes.

### 2.3 Oral production and the differential effect on speaking

Explanation of the reason for the effect to focus on speaking and be uneven can be found in the process of oral communication. Interaction allows learners to acquire a second language by means of meaning negotiation, where they indicate that the message failed to get across and make changes in order to improve comprehension (Long, 1996). Moreover, interaction makes it possible for learners to practice formulating their output (Swain, 1995). Not all sub-skills of oral production emerge at the same time. With limited attentional capacity, students who participate in communication-oriented activities focus more on meaning than on form, and thus improvement in the sub-skills of fluency and interaction occurs earlier than the sub-skills related to grammar or complexity. There is also the possibility of deterioration in some dimensions (Hasnain and Halder, 2024). It is unlikely that any short-term classroom intervention can improve all dimensions equally, while the dimensions that respond better to motivation and interaction are those of fluent, contingent communication.

*H3a*: The project-based group makes greater gains in fluency than the control condition.

*H3b*: The project-based group makes greater gains in pragmatic and interactional competence than the control condition.

*H3c*: The project-based group’s advantage over the control condition is greater for fluency and pragmatic-interactional competence than for accuracy and complexity.

### 2.4 The serial mediation model

These two processes discussed above not only define two variables associated with speaking but also serve another purpose beyond that. From the perspective of self-determination theory, autonomous motivation represents the immediate psychological outcome of need support in that context, thereby marking the point where external factors are transformed into self-motivated behavior. Similarly, collaborative learning views engagement as the behavioral mechanism by which such behavior becomes performance. Therefore, motivation and engagement are mediators rather than two separate outcomes, and the effect of project-based learning on the distal skill of speaking takes place via these mediators rather than directly. In mediation studies conducted on the same sample group, the view is confirmed by the finding that basic psychological needs and engagement mediate the effect of a supportive environment on oral achievement (Wang et al., 2024) and peer support and engagement mediate the effect of collaboration in college English (Li, 2025).

Both motivation and engagement are parts of a chain, which are not inter-changeable, but sequential. Autonomous motivation acts as the main factor driving cooperation, because only motivated students will be involved in cooperative activities. Otherwise, cooperation, not being driven by motivation, can easily dissipate. Consequently, motivation comes before and fuels engagement, while engagement provides the interactional behavior that facilitates speech production. This reasoning leads to the sequential model below, where the intervention raises motivation, which promotes engagement, and the latter positively affects oral performance (Figure 1).

**Figure 1:**
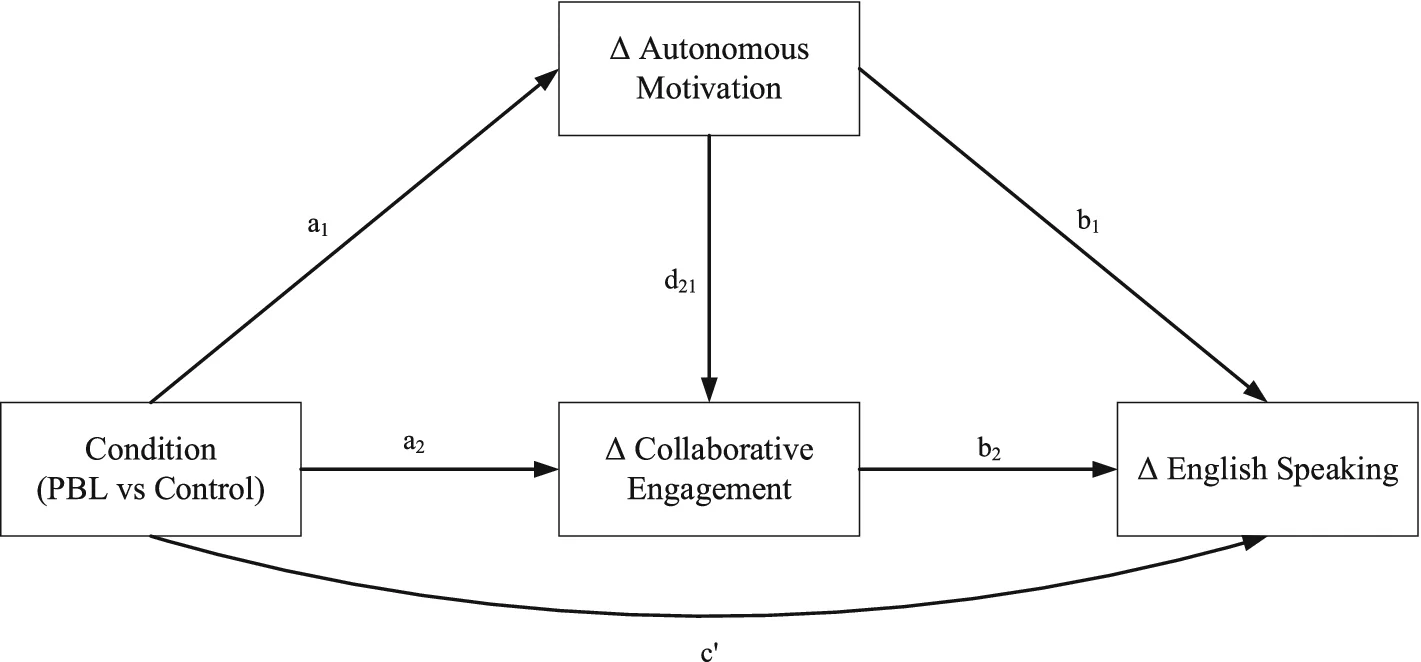
Hypothesized serial mediation model. H1 corresponds to a₁; H2 to a₂; H4a to a₁ × b₁; H4b to a₂ × b₂; H4c to the serial indirect path a₁ × d₂₁ × b₂. H3 (differential sub-dimension pattern) is tested across separate mediation models for the composite, fluency, and pragmatic-interactional outcomes rather than on a single path.

*H4a*: Autonomous motivation mediates the effect of project-based learning on speaking competence.

*H4b*: Collaborative engagement mediates the effect of project-based learning on speaking competence.

*H4c*: Autonomous motivation and collaborative engagement serially mediate the effect of project-based learning on speaking competence, with motivation preceding engagement.

## 3 Materials and methods

### 3.1 Design and procedure

The design used for this research was quasi-experimental and sequential explanatory mixed method (Creswell and Clark, 2017). Two full College English classes at the institution were assigned to either the experimental group using the project-based learning (PBL) method or the control group using traditional methods of teaching. Data collection for this research was conducted at three separate time intervals. The first time point (T1) was a pretest measured 1 week prior to the start of instruction, the second time point (T2) was an immediate posttest measured 1 week after the 12-week instructional period, and the third time point (T3) was a delayed posttest 6 weeks after T2. The quantitative data gathered per wave involved the autonomous motivation and collaborative engagement questionnaires as well as a paired task speech sample evaluated by two raters. The qualitative data was obtained at T2 through semi-structured interviews and classroom observation.

### 3.2 Participants and setting

A total of 240 second-year students enrolled at a private higher vocational college (City University of Zhengzhou) in Zhengzhou, Henan Province, central China participated in the study and satisfied the inclusion criteria, out of which 120 were put into the PBL group and 120 into the control group. The inclusion criteria consisted of attending the College English class, being a native speaker of the Mandarin language, intermediate proficiency in the English language (B1 level according to CEFR on institutional placement), and signing the consent form. The exclusion criteria consisted of having any learning or language disabilities, over 6 months spent studying abroad in an English-speaking country, and having taken part in an English project-based course before. For estimation of serial mediation using bootstrap confidence intervals that are bias corrected, the analytical minimum of 150 cases per small-to-medium indirect effects was considered, with the target sample size ranging from 180 to 200 cases (Fritz and MacKinnon, 2007). The problems associated with dropout-induced missing data were managed by conducting complete-case analysis where the subjects who completed the test and those who did not were compared on various baseline measures. The study was approved by the Academic Board of City University of Zhengzhou, a private higher vocational college in Zhengzhou, Henan Province, China (approved on 20 August 2025; valid through 19 August 2026). Participation was voluntary, written informed consent was obtained, data were anonymized at source, and participants could withdraw at any point without academic consequence.

### 3.3 The PBL intervention and the control condition

The intervention took place during a span of 12 consecutive weeks, during the timeframe of nine to eighteen weeks that are known to be linked with increased PBL effectiveness (Zhang and Ma, 2023). PBL learners participated in teams comprising four to five members on three sequential assignments, which were community issue research, workplace scenario, and documentary type of presentation. There were different stages involved in each of the projects including the driving question stage, planning and division of roles, research in English and writing, feedback from peers, rehearsals, and the production stage. The teacher was the facilitator who offered scaffolding through milestone check-ins without engaging in any form of lectures. The control group used the traditional textbook approach involving teacher explanation and controlled practice, followed by individual activities. The two groups covered identical vocabulary and grammatical structures based on common lesson plans, and both were taught by teachers with comparable experience levels. Implementation fidelity was assessed by making weekly observations of the class and recording the proportion of class time spent on teacher-led, individual, and group-based activities.

### 3.4 Instruments and data collection

#### 3.4.1 Quantitative measures

Autonomous motivation was assessed through the eight questions about autonomous regulation of the Learning Self-Regulation Questionnaire (SRQ-L) (Williams and Deci, 1996) on a seven-point scale (1 = not at all true, 7 = very true). Collaborative engagement was assessed by the 10-item measure constructed out of previously validated scales measuring collaboration in college settings (Li, 2025), rated on the same seven-point scale. The questionnaires were translated to Chinese language and back-translated to English by two bilingual researchers (Brislin, 1970), and the differences that arose were sorted out before the administration of the questionnaire.

The participants’ English-speaking competence was measured using a paired-part task model including a topic-card monolog and a discussion based on the IELTS Speaking format, where an analytic scoring rubric was used in a nine-band scale. The four criteria were assessed in a nine-band scale in half-band units: fluency, accuracy, complexity, and pragmatic-interactional competence. The performances were rated by two raters that possessed credentials to teach English separately, independent of condition and wave. There were three calibration sessions conducted on benchmark performances for rater training, and any differences exceeding one band were reconciled before actual rating. The final score on each dimension was an average of the two raters’ bands.

#### 3.4.2 Qualitative data

Following T2, 24 students were selected using purposeful sampling techniques and interviewed using semi-structured interviews meant to capture the spectrum of oral gains made by the students in different conditions and groups. There were 12 students per condition. The interview schedule began with an open recall of any incidents where oral progress had been perceived and moved on to probes on group processes such as free-riding, dominance, and silence. The interviews lasted from 25 to 40 min and were audio-taped with the prior permission of the respondents. Along with the interviews, field notes of the observations conducted in the PBL condition, including 12 class observations altogether, were also used as the source of information for the study. The analysis of transcripts was conducted through reflexive thematic analysis using the six-stage process described by Braun and Clarke (2006), where two analysts coded the transcripts using NVivo 12.

### 3.5 Data analysis

Quantitative analysis was conducted using SPSS 26. The tests for univariate normality were performed through skewness, kurtosis, and the Shapiro–Wilk’s test while the test for homogeneity of variance was conducted using Levene’s test. Tests for sphericity were performed using Mauchly’s test followed by Greenhouse–Geisser correction where appropriate. The equivalence at the baseline of the two conditions was established via independent samples t-tests on the T1 scores and chi-square tests for categorical demographics. For all constructs, the effects were tested using mixed design analyses of variances (condition × wave), with partial eta-squared being stated for all effects. All interactions of significance were determined by Bonferroni-adjusted pair-wise tests among waves under each condition. Group differences in terms of changes from T1 were given in terms of Cohen’s d effect size measure. Inter-rater reliability in relation to the speaking rating scale was assessed through the use of the intra-class correlation coefficient (ICC [2,1]). Retention from T2 to T3 was computed as the ratio of the T3 gain to the T2 gain.

To test for serial mediation effects, Model 6 from the PROCESS macro was used (Hayes, 2017). Condition was treated as the predictor, the change from T1 in autonomous motivation as the first mediator, the change from T1 in collaborative engagement as the second mediator, and the change from T1 in the relevant speaking outcome as the dependent variable. A total of 5,000 bootstrap resamples were taken, and 95% confidence intervals adjusted for bias were computed. Three dependent variables were measured simultaneously: the speaking scale, fluency, and pragmatic interactional skill. Maintenance of treatment effects between T2 and T3 was evaluated as the percentage of the T2 effect maintained at T3 and as the Cohen’s d difference. Quantitative and qualitative results were integrated narratively, with qualitative themes mapped onto the corresponding quantitative findings.

## 4 Results

### 4.1 Preliminary analyses

There were 240 participants in the study, of whom 179 (74.6%) successfully completed all three measurement waves: 93 out of 120 in the PBL condition (drop-out rate = 22.5%), and 86 out of 120 in the control condition (drop-out rate = 28.3%). No differences between completers and drop-outs were detected on any baseline measure (all *t* < 1.0, all *p* > 0.36) or on condition, gender and age (all *p* > 0.37), thus indicating a missing-completely-at-random pattern. There was no difference between the two conditions at baseline on any demographic characteristic or outcome measure (all *p* > 0.14, all |d| < 0.23). The distribution by gender was as follows: females = 63/54; males = 30/32 in the PBL and control conditions, respectively; *χ*^2^(1) = 0.29, *p* = 0.590. Distribution by major was as follows: business = 28/17; engineering = 21/22; humanities = 14/17; science = 19/21; education = 11/9 in the PBL and control conditions, respectively; *χ*^2^(4) = 3.03, *p* = 0.552. Age was similar in both conditions [*M* = 20.12, SD = 1.09/*M* = 20.15, SD = 1.02 in the PBL and control conditions, respectively; *t*(177) = −0.21, *p* = 0.836]. Implementation fidelity was high, as shown by the classroom observations. For example, in the PBL condition, class time was allocated in the ratio of 14.8% teacher fronted, 21.3% individual, and 63.9% group activity. In the control condition, it was 58.4%, 31.2%, and 10.4%, respectively.

Skewness and kurtosis for all outcome variables fell within ±2; although Shapiro–Wilk reached significance in a few cases, the minor deviations and large sample supported the use of parametric analyses. The homogeneity of variances assumption at T1, which was tested using Levene’s test, was satisfied for all dependent variables (all *p* > 0.10). The assumption of sphericity was violated in autonomous motivation, fluency, complexity, pragmatic-interactional competence, and the overall speaking score, as revealed by Mauchly’s test of the wave factor (Mauchly *p* from < 0.001 to < 0.01). Greenhouse–Geisser adjustment for the within-subject effect was used for these variables. Cronbach’s α coefficients were adequate for both instruments across waves (Table 1). At T1, autonomous motivation correlated significantly with collaborative engagement (*r* = 0.59) and composite speaking (*r* = 0.67); and collaborative engagement correlated significantly with composite speaking (*r* = 0.71), all *p* < 0.001.

**Table 1:** Descriptive statistics and internal-consistency reliability (completers, *N* = 179).

Variable	T1 *M* (SD) PBL	T1 *M* (SD) CTL	T2 *M* (SD) PBL	T2 *M* (SD) CTL	T3 *M* (SD) PBL	T3 *M* (SD) CTL	*α* T1/T2/T3
Autonomous motivation	4.07 (0.78)	4.19 (0.81)	4.71 (0.90)	4.33 (0.93)	4.64 (0.95)	4.52 (0.92)	0.84/0.88/0.88
Collaborative engagement	3.96 (0.87)	4.08 (0.74)	4.68 (0.86)	4.25 (0.93)	4.52 (1.04)	4.35 (0.92)	0.87/0.90/0.91
Fluency	4.88 (0.77)	4.93 (0.90)	5.91 (1.01)	5.20 (1.06)	5.84 (1.08)	5.32 (0.99)	–
Accuracy	5.07 (0.80)	5.19 (0.79)	5.36 (0.95)	5.29 (0.87)	5.42 (0.90)	5.27 (0.74)	–
Complexity	4.66 (0.82)	4.83 (0.77)	4.97 (0.90)	4.80 (0.83)	4.94 (0.84)	4.88 (0.94)	–
Pragmatic-interactional	4.96 (0.84)	4.92 (0.87)	5.79 (0.95)	5.13 (0.92)	5.65 (0.93)	5.16 (0.95)	–
Composite speaking	4.89 (0.67)	4.97 (0.71)	5.51 (0.62)	5.10 (0.58)	5.46 (0.55)	5.16 (0.43)	–

### 4.2 Effects on autonomous motivation and collaborative engagement

Interaction of Group × Wave on AM was significant, *F*(2, 354) = 6.62, *p* = 0.002, partial *η*^2^ = 0.036 (Table 2), suggesting a medium between-group effect on change from T1 to T2 (*d* = 0.57) and a small effect at T3 (*d* = 0.24). A similar pattern was found for CE, *F*(2, 354) = 7.72, *p* < 0.001, partial *η*^2^ = 0.042, *d* = 0.64 at T2 and *d* = 0.28 at T3. From *post hoc* tests, the increase was significant between T1 and T2 in both constructs for the PBL group (*t* = 6.81 and *t* = 8.03; both *p* < 0.001) but there was no significant increase between T2 and T3, while the control group had no significant increase between T1 and T2. Therefore, H1 and H2 are supported.

**Table 2:** Mixed-design analyses of variance for autonomous motivation and collaborative engagement.

Variable	Source	SS	df1	df2	MS	*F*	*p*	Partial *η*^2^
Autonomous motivation	Group	2.10	1	177	2.10	1.41	0.237	0.008
	Wave	21.84	2	354	10.92	25.83	< 0.001	0.127
	Group × Wave	5.59	2	354	2.80	6.62	0.002	0.036
Collaborative engagement	Group	3.35	1	177	3.35	2.13	0.146	0.012
	Wave	22.78	2	354	11.39	26.45	< 0.001	0.130
	Group × Wave	6.65	2	354	3.32	7.72	< 0.001	0.042

df and partial *η*^2^ are reported uncorrected; p-values for within-subjects effects are adjusted with the Greenhouse–Geisser correction where Mauchly’s test indicated a sphericity violation.

### 4.3 Effects on speaking by sub-dimension

Spoken language growth showed different trajectories according to each of the four rubric dimensions, as illustrated in Figure 2. Interaction effect of Group and Wave attained statistical significance and a large effect size for fluency, *F*(2, 354) = 15.15, *p* < 0.001, partial *η*^2^ = 0.079, and pragmatic-interactional competence, *F*(2, 354) = 10.00, *p* < 0.001, partial *η*^2^ = 0.053. The interaction was statistically non-significant for accuracy, *F*(2, 354) = 2.10, *p* = 0.124, partial *η*^2^ = 0.012, and statistically significant but small for complexity, *F*(2, 354) = 3.71, *p* = 0.025, partial *η*^2^ = 0.021. Interaction of Group and Wave for the composite speaking performance was highly significant, *F*(2, 354) = 21.83, *p* < 0.001, partial *η*^2^ = 0.110. The standardized effect sizes of between-group differences in change scores were consistent with expectations as follows: *d* = 0.82 in fluency and d = 0.68 in pragmatic-interactional competence in comparison with *d* = 0.23 in accuracy and *d* = 0.48 in complexity. The PBL group significantly surpassed its peers in relation to fluency and pragmatic-interactional competence to a much greater extent than it did in accuracy and complexity, therefore confirming the differential impacts in H3a, H3b, and H3c. Inter-rater reliabilities across time periods and constructs ranged from ICC(2,1) = 0.76 to 0.87 (Table 3). The effect size of change measured on the composite level (*d* = 1.07 at T2) was higher than d of all the constructs, since the standard deviation of change scores of the composite (0.45) was lower than that of all the constructs (0.73–0.93). This is because taking an average of the four dimensions decreased the variance on account of errors from both raters and items being uncorrelated. Furthermore, the decrease was further facilitated by the extremely low correlation among the dimensions (*r* = 0.02 to 0.16).

**Figure 2:**
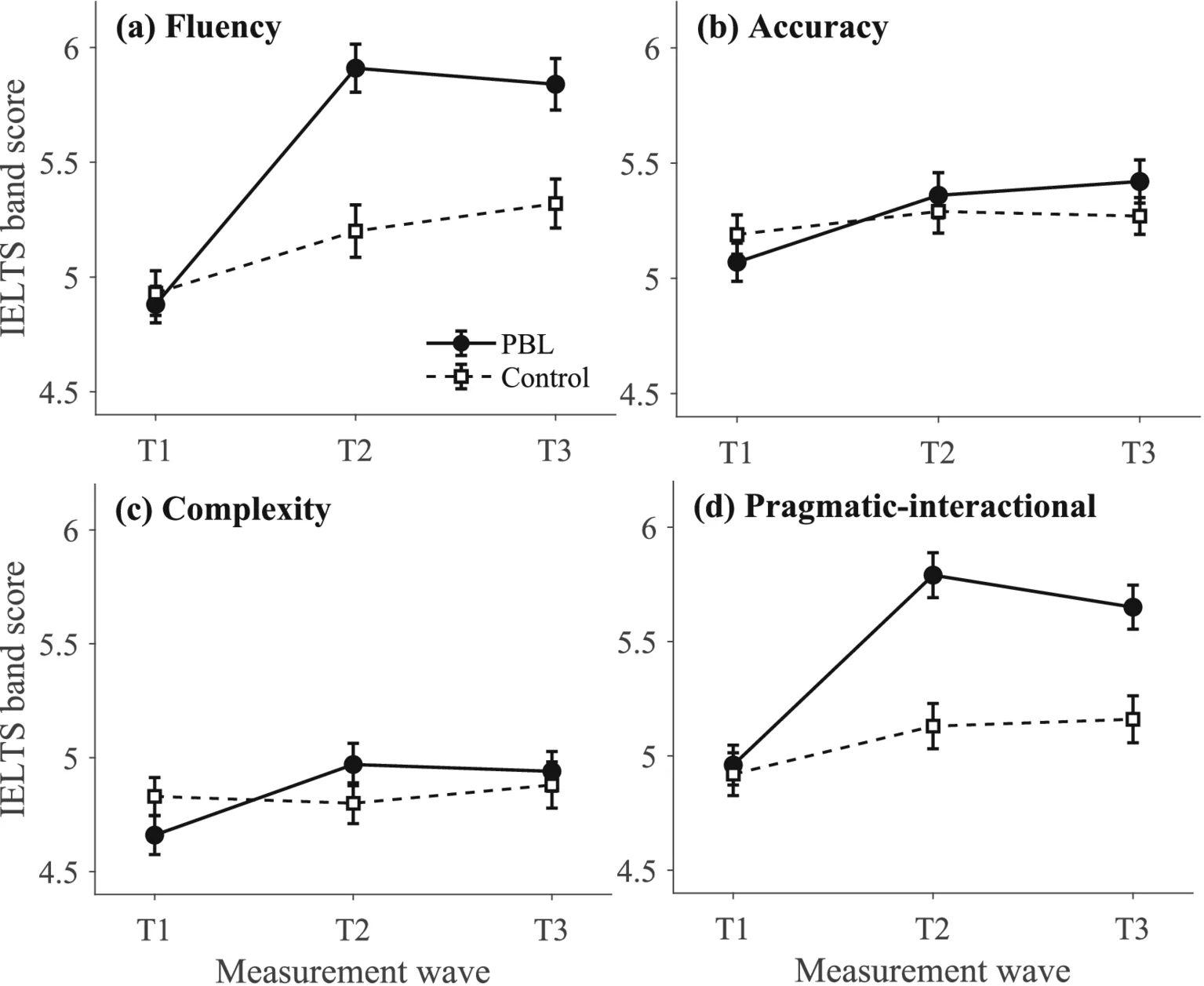
Trajectories of the four speaking sub-dimensions across T1, T2, and T3 by condition. **(a)** Fluency, **(b)** Accuracy, **(c)** Complexity, **(d)** Pragmatic-interactional competence.

**Table 3:** Inter-rater reliability for the analytic speaking rubric across waves.

Dimension	T1 ICC(2,1)	T2 ICC(2,1)	T3 ICC(2,1)
Fluency	0.76	0.87	0.87
Accuracy	0.78	0.80	0.78
Complexity	0.76	0.81	0.78
Pragmatic-interactional	0.79	0.83	0.83

### 4.4 Mediation

Regarding the composite measure for the change in speaking, the total effect of the condition was *b* = 0.48, 95% CI [0.35, 0.62], and the direct effect was still significant after the mediators were controlled for (*b* = 0.42, 95% CI [0.29, 0.56]), which indicated partial mediation (see Table 4). Specifically, only indirect effects through engagement (*b* = 0.075, 95% CI [0.024, 0.145]) were found significant, and indirect effects through motivation alone were not (*b* = −0.025, 95% CI [−0.068, 0.009]). The serial indirect effects (condition → motivation → engagement → speaking) had a significant impact (b = 0.014; 95% CI = 0.002–0.031), which is illustrated in Figure 3. In addition, fluency as well as changes in pragmatic-interactional competence showed the same pattern whereby the serial and engagement-only indirect effects were significant but not motivation-only effect. The results support the hypotheses H4b on all three dependent variables, H4c on the issue of serial mediation, and contribute to the specification of hypothesis H4a because the effect of autonomous motivation on speaking was fully mediated by collaboration.

**Table 4:** Bootstrapped direct and indirect effects of condition on speaking outcomes (5,000 resamples, bias-corrected 95% CI).

Outcome	Path	Estimate	95% CI
Composite speaking	Total (c)	0.48	[0.35, 0.62]
	Direct (c′)	0.42	[0.29, 0.56]
	Indirect via AM	−0.03	[−0.07, 0.01]
	Indirect via CE	0.07	[0.02, 0.14]
	Serial: AM → CE	0.014	[0.002, 0.031]
Fluency	Total	0.77	[0.48, 1.03]
	Direct	0.67	[0.38, 0.94]
	Indirect via AM	−0.02	[−0.11, 0.06]
	Indirect via CE	0.10	[0.02, 0.20]
	Serial: AM → CE	0.019	[0.002, 0.049]
Pragmatic-interactional	Total	0.63	[0.36, 0.90]
	Direct	0.52	[0.23, 0.80]
	Indirect via AM	−0.03	[−0.11, 0.04]
	Indirect via CE	0.12	[0.03, 0.24]
	Serial: AM → CE	0.022	[0.003, 0.051]

AM = autonomous motivation; CE = collaborative engagement; condition coded PBL = 1, control = 0.

**Figure 3:**
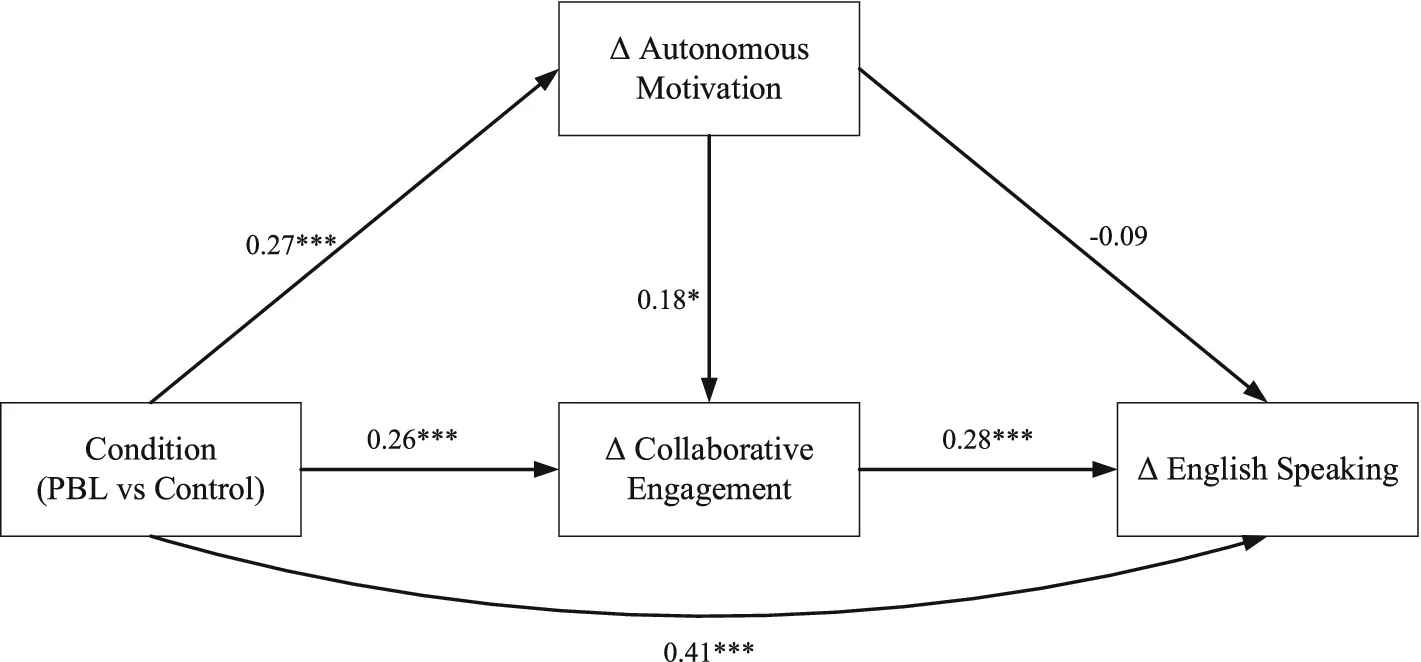
Empirically tested serial mediation model for composite speaking with standardized path coefficients. ***p < 0.001, **p < 0.01, *p < 0.05.

### 4.5 Maintenance at delayed post-test

The retention rate of PBL gains in T3 varied for all constructs (Table 5). Autonomous motivation retained 88% of its T2 gain at T3; the collaboration gains remained at 78%; fluency gains were retained at 94% while pragmatic-interactional gains were retained at 84%. Accuracy and complexity gains were initially small and they either retained or slightly increased. For the d values of the speaking composite scores, there was a decrease from 1.07 at T2 to 0.63 at T3, for fluency (0.82 to 0.53), and pragmatic-interactional skills (0.68 to 0.42). The within-PBL T2-T3 differences for all outcome measures failed to reach statistical significance (all *p* > 0.08), suggesting that the progress made at T2 was largely maintained after 6 weeks.

**Table 5:** Maintenance of PBL gains from T2 to T3.

Variable	PBL gain T2 (T2 − T1)	PBL gain T3 (T3 − T1)	Retention rate	d at T2	d at T3
Autonomous motivation	0.641	0.565	88%	0.57	0.24
Collaborative engagement	0.716	0.555	78%	0.64	0.28
Fluency	1.027	0.962	94%	0.82	0.53
Accuracy	0.293	0.352	120%	0.23	0.28
Complexity	0.309	0.277	90%	0.48	0.23
Pragmatic-interactional	0.828	0.694	84%	0.68	0.42
Composite speaking	0.614	0.571	93%	1.07	0.63

### 4.6 Qualitative findings and integration

Four themes have emerged from the reflexive thematic analysis conducted on the data from 24 interviews and 12 observations regarding mechanisms, and three themes related to negative dynamics (Table 6). The inter-coder reliability was 86% on the code level and 92% on the thematic level. Ownership as a mechanism of autonomy, output in preparation for public display as a mechanism of competence, collaboration as a mechanism of relatedness, and interaction pressure as a mechanism of engagement are some mechanisms which correspond to the quantitative finding that it is engagement and not motivation which transfers the impact of the condition on speaking. Participants attributed their willingness to speak to the accountability built into group work rather than to personal motivation. The presence of negative dynamics such as free riding, dominance in discourse, and the instruction language turning toward the Chinese language may account for why there has been little improvement in accuracy and complexity within the PBL condition due to the reduction of attention-consuming production.

**Table 6:** Qualitative themes, definitions, and illustrative excerpts.

Theme	Definition	Illustrative excerpt
Autonomy through ownership	Choice over topic, role, and pacing within the project	“We chose to do food delivery in our city because we actually wanted to know how it works, not because the teacher told us to.”
Competence through visible output	Confidence built as drafts became a polished, audience-ready oral product	“The first rehearsal was awful, but by the third one I knew exactly what to say, and that gave me the courage to speak in front of the class.”
Relatedness through extended collaboration	Sustained groupwork strengthened peer bonds	“After working together for so many weeks, I stopped being embarrassed to make mistakes in front of them.”
Engagement through interactional pressure	Group accountability obliged speaking that would not occur in solo work	“If I did not say my part, the others could not move on, so I had to speak even when I wasn’t ready.”
Free-riding	Less proficient members deferred effort and speaking turns to stronger peers	“Two members did most of the talking. I just smiled and added a sentence at the end.”
Conversational dominance	One or two proficient students took over turn space in planning and rehearsal	“She always corrected us before we finished, so after a while I stopped trying to say long sentences.”
L1 slippage	Planning drifted into Chinese, reducing English production	“When we needed to decide quickly, we just switched to Chinese. We only used English for the final presentation.”

## 5 Discussion

This sequence indicates that there is some kind of psychological mediation of the proximal variables between the PBL process and distal language acquisition as an outcome of that process. In the case of the experimental group, it should be stated that the autonomous motivation and the collaboration were observed at once after the intervention, and the mediated effect of engagement had a considerable impact on the composite, fluency, and pragmatic-interactional outcomes. Speaking was not achieved directly by motivation. In contrast, it was through engagement that motivation operated. It is further made clear through the findings from retention that the timing of the process took place. The proximal mediators retained 88 and 78%, respectively, of their immediate influence after 6 weeks of training while the distal outcome retained 93%. Hence, there was greater loss for the mediators than for the distal outcome. The process of sequential mediation is well-aligned within the theoretical perspective of self-determination theory (Ryan and Deci, 2000). Engagement mediates the relationship between psychological need antecedents and speaking of English in class (Wang et al., 2024). The current research makes a contribution by incorporating a four-dimensional rater-judged assessment, a delayed retention measurement, and an experimental contrast to that earlier cross-sectional model. While the existence of the positive link between classroom engagement and PBL (Zhong et al., 2025) does not indicate causation per se with respect to speaking performance, it is established through the sequence of the mediational process within the present design. Speaking enjoyment and behavioral engagement were positive predictors of willingness to communicate (Lin and Wang, 2025). The connection between classroom interaction and willingness to communicate was mediated by speaking self-efficacy and enjoyment (Liu et al., 2025). Collectively, these consistent results provide evidence that the process of engagement and interaction works within the framework of the outcome measure of communication irrespective of its difference from the measure of performance adopted in this study. The reasoning from the proximal to distal approach makes the present study distinguish from the purely outcome-oriented evaluation of PBL (Zhang and Ma, 2023) and non-retention tests which failed to differentiate between temporary and long-term effects of PBL.

The four sub-dimensions of speaking were not equally affected during their development by the PBL treatment. Speaking sub-dimensions benefit greatly from an advantageous condition in fluency and pragmatics interaction. There is also a small advantage for complexity and a non-advantageous condition in accuracy. Such an arrangement suits well the output hypothesis and interaction hypothesis (Long, 1996; Swain, 1995). As the task needed many opportunities for production of speech and interaction (working in groups, preparing drafts, giving presentations), it made it possible for the students to improve the components of speaking which can be acquired due to frequent production and turn-taking. The development of accuracy and complexity is controlled by some other factors, where the former is related to the aspect of form focus and correction and the latter by planning time and conscious encoding, both of which were not addressed in the current project. The idea of L2 speech production being limited by the attention span requires that learners manage the various complexity, accuracy, and fluency (CAF) elements in a way that allows them to communicate effectively. It is stated that improvement in one of the CAF categories may occur at the expense of the other because there is an inverse relationship among them (Li and Sui, 2025). Task-based instruction has the benefit of promoting fluency and complexity, but not accuracy in comparison with the presentation-practice-production model (Lu et al., 2025). For the short-term, non-tertiary PBL experiment, motivational improvement existed alongside insignificant results on comprehensive achievement tests and a lack of separation into oral skills (Yang et al., 2025). This difference between the cited research and the current results seems to be due to the interaction of the intervention time, target population, and outcome measurement. Therefore, the difference pattern cannot be treated as the limitation of PBL, but it should be regarded as the precise explanation of the method.

Speaking was most affected indirectly by collaborative engagement of the three mediation frameworks studied. This finding was validated through the qualitative findings, where speaking apart from personal involvement was attributed to group accountability. The mechanism through which this occurred was identified as group pressure for participation, and peer accountability led to increased engagement during group activities (Adesina et al., 2023). It should be noted that certain factors were identified in the qualitative data which weakened the impact, including free riding by lower-proficiency participants, dominance in discussion by higher-proficiency individuals, and a shift from the group interaction language to Chinese due to time pressure. The latter factor is consistent with the discovery that the degree of L1 utilization during dyadic speech is determined by the proficiency pairs and tasks (Tian and Jiang, 2021). Larger groups are more prone to turn-taking asymmetry, consistent with the finding that smaller groups promote peer interaction more than larger ones (Wang et al., 2023). The process whereby speaking contributes to the benefit of the group is structured in such a way that, depending on some circumstances, it can hinder the benefit of the individuals.

### 5.1 Implications of the study

That motivation reached speaking only once it had been converted into collaborative behavior bears on how motivational accounts of second language speaking should be specified. Need satisfaction is better modeled as a necessary but insufficient antecedent of oral gains, with the behavioral step of sustained peer interaction supplied in between, so that models predicting speaking directly from motivational states remain underspecified. The retention asymmetry sharpens this reading, since gains that were no more than the trace of a temporarily elevated motivational state should have decayed at least as fast as that state itself. What the intervention left behind is therefore better described as consolidated skill, and the mediators as the conditions under which practice takes place rather than as the gains themselves.

The designable ingredient of project-based learning, on this evidence, is the accountability structure of the group rather than the choreography of the task. That structure should be engineered directly, through individually traceable speaking roles rotated across project cycles, explicit turn allocation during planning and rehearsal, groups kept toward the smaller end of the range used here, and free-riding monitored through peer assessment of the collaborative process rather than of the final product alone (Adesina et al., 2023). The drift into Chinese under time pressure is better met by a protocol that concentrates L1 use at designated decision points than by a blanket English-only rule. Because the sub-dimension pattern was uneven, such programs should not be evaluated against accuracy-weighted instruments, and short form-focused episodes are best placed at the rehearsal stage, where output has already been drafted and attention is no longer fully absorbed by meaning negotiation. Given the decay observed at the mediator layer, recurring project cycles distributed across semesters are more likely to sustain these conditions than a single project module.

### 5.2 Limitations and future research

Several important limitations apply to the design. The sampling of intact groups from a single private higher vocational college in Zhengzhou, Henan Province, China makes the results applicable only to comparable higher vocational institutions and to intermediate-proficiency (B1) learners, rather than to research-intensive universities or to markedly lower- or higher-proficiency populations. Additionally, since the design is not randomized, it cannot attain the inferential power of randomized experiments, despite the baseline equivalence. Traditional, teacher-led textbook instruction was used as the control condition. Hence, the experimental outcomes reflect the influence of PBL in contrast to the traditional method, although they cannot specify the specific benefits of PBL compared to other learner-centered approaches, such as task-based or content-based English language instruction. The six-week delayed post-test captures short-term retention only; longer-term effects would need to be assessed over a longer horizon or through out-of-class communication. Engagement was measured by self-report, and qualitative observation supplements but does not substitute for a behavioral measure of class participation. Further research might widen the sample to encompass more higher vocational colleges, extend the intervention across a full academic year, and code classroom participation from video footage. It might also examine whether the collaborative scaffolding effect operates differently for low-proficiency students, for whom the negative aspects of group work may be most pronounced.

## 6 Conclusion

With respect to this quasi-experimental study based on mixed methodology, the pathway through which PBL impacts speaking competence in EFL learning environment at Chinese higher vocational colleges has been identified. It is claimed that the pathway through which the impact occurs begins with the arousal of autonomous motivation and leads to collaboration, through which the impact is made. Four-dimensional assessment based on rater judgment helps further clarify the above hypothesis with regard to how PBL improves the speaking process using the skill-specific model whereby the influence on fluency and interaction is significant, the influence on complexity is small, and the influence on accuracy is not statistically detectable. A six-week delay of measurement sets apart the more decay-vulnerable mediator layer (88% of motivation retention, 78% of engagement retention) from the more stable outcome layer (93% of composite speaking retention), and thus an acquisition approach is preferred to the transient activation approach. The pedagogical implication is that the active ingredient of PBL is the psychological condition it creates rather than the task sequence itself, so instructional design should be judged by how well it sustains learners’ autonomy, competence and relatedness, with task structure and form-focused work supporting those conditions rather than substituting for them.

## Data Availability

The original contributions presented in the study are included in the article/Supplementary material, further inquiries can be directed to the corresponding author.
